# Dynamics of a Sex-Linked Deleterious Mutation in Populations Subject to Sex Reversal

**DOI:** 10.1371/journal.pone.0025362

**Published:** 2011-10-10

**Authors:** Markku Karhunen

**Affiliations:** Department of Biosciences, University of Helsinki, Helsinki, Finland; British Columbia Centre for Excellence in HIV/AIDS, Canada

## Abstract

The heterogametic sex chromosomes (i.e. mammalian *Y* and avian *W*) do not usually recombine with the homogametic sex chromosomes which is known to lead into rapid degeneration of *Y* and *W* due to accumulation of deleterious mutations. On the other hand, some 96% of amphibian species have homomorphic, i.e. non-degenerate 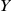 chromosomes. Nicolas Perrin's fountain-of-youth hypothesis states that this is a result of recombination between 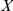 and 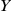 chromosomes in sex-reversed individuals. In this study, I model the consequences of such recombination for the dynamics of a deleterious mutation occurring in 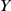 chromosomes. As expected, even relatively low levels of sex reversal help to purge deleterious mutations. However, the population-dynamic consequences of this depend on the type of selection that operates on the population undergoing sex reversal. Under fecundity selection, sex reversal can be beneficial for some parameter values, whereas under survival selection, it seems to be always harmful.

## Introduction

Some recombination needs to occur between homologous chromosomes to enable proper pairing during meiosis. In heterogametic sex chromosomes (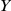 or 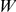 for male or female heterogamety, respectively), this recombination occurs only in so-called pseudoautosomal regions. Typically, the pseudoautosomal regions are relatively small, and as a result of this, sex chromosomes missegregate more frequently than autosomes [1]. It is thought that large non-recombining regions have evolved because of the need to maintain linkage between sex-determining loci and other loci which are subject to sexual-antagonistic selection [2]. Presumably as a result of this, the heterogametic sex chromosomes are often ‘degenerate’, i.e. they have fewer functional genes than the homogametic sex chromosomes [3], [4], [5].

Charlesworth and Charlesworth [3] provide an excellent review of how the 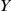 chromosomes degenerate. To start with, deleterious mutations occurring in the non-recombining regions of 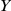 chromosomes are always heterozygous, and selection against them is weaker, accordingly. Secondly, the effective population size of 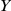 chromosomes is only one third of that of 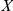 chromosomes, and one fourth of that of autosomes, and thus drift effects are more important in case of 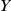 chromosomes. The consequences of a weakened selection and a reduced effective population size are manifold. Charlesworth and Charlesworth [3] discuss four main effects: Firstly, Muller's ratchet [6] implies that deleterious mutations may reach fixation purely as a result of random drift, turning off one gene after another. In 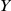 chromosomes, this fixation process is much faster than in freely recombining chromosomes. Secondly, mildly deleterious mutations may hitchhike into fixation when they are linked with beneficial mutations [7]. Thirdly, the need to purify the genome of strongly deleterious mutations may drive mildly deleterious ones into fixation, if they are in negative linkage with the more deleterious ones [8]. Finally, Hill-Robertson effects [9] may cause that new, functional variants are unable to rejuvenate the 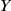 chromosomes, because they usually occur in different haplotypes, which causes a diversifying selection pressure.

However, not all 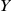 chromosomes are equally degenerate. For example, in amphibians, the vast majority of species have homomorphic sex chromosomes, so that the 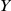 and 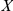 chromosomes are indistinguishable on basis of the karyotype [10]. It looks that there has to be some mechanism counter-acting the decay of these 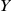 chromosomes [10], [11], [12]. One explanation could be a high turnover rate: new sex-determining loci may appear on autosomes so rapidly that the 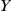 chromosomes do not have time to decay [13]. Another possibility is that 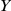 chromosomes recombine more than has been previously thought. In humans, it is likely that duplications and 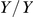 gene conversions help to prevent the decay of the 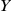 chromosome, and this may also be the case of other mammalian species [14], [15]. Even recombination between 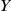 chromosomes and autosomes has been described [16].

Nicolas Perrin's fountain-of-youth hypothesis [10] states that in certain species, 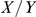 recombination help to maintain the 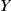 chromosomes. This concerns recombination outside the pseudoautosomal regions which is thought to occur in sex-reversed females (i.e. 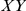 females). This hypothesis rests on two cornerstones: Firstly, 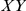 females need to be produced by some mechanism, which we will refer to as ‘sex reversal’. Secondly, it has to be assumed that the recombination pattern depends on the phenotypic sex.

Concerning the plausibility of Perrin's hypothesis [10], it is known that sex-reversed individuals (i.e. 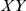 females and 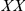 males) occur in considerable quantities in many species of fish, amphibians and reptiles [17], [18], [19], [20], [21], [22]. In fact, a pure genetic sex determination system where 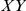 individuals are bound to be males, is a special case in the animal kingdom, and no more ‘natural’ than an environmental sex determination system [23]. In addition, the rate of recombination, both in autosomes and sex chromosomes, typically depends on the sex of the individual. This pattern is known as ‘heterochiasmy’ [24]. Specifically, there is a growing body of evidence indicating that the recombination rate depends on the phenotypic (instead of the genotypic) sex [25], [26], [27], [28], [29]. Why this is the case, is at present unknown, but differences in the physiological process of male and female meiosis have been offered as an explanation [30]. Secondly, sex-specific differences in gene regulation may play a role [10].

In this study, I provide a mathematical demonstration of Perrin's fountain-of-youth hypothesis. To this end, I develop analytical theory by writing a dynamical system for allele frequencies in sex chromosomes of populations that are subject to sex reversal. Secondly, I test the predictions of this theory by using individual-based simulations.

## Methods

### Modeling framework

I consider three loci in sex chromosomes, denoted by 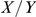, 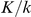 and 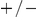. Locus 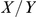 determines the genotypic sex which can be environmentally reversed. Locus 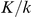 is subject to sexual-antagonistic selection such that 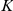 alleles (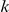 alleles) are subject to a selection differential 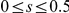 in phenotypic males (females), i.e. it is harmful to inherit alleles that are adapted for the other sex. This represents the fitness cost associated with sex reversal. Locus 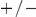 is subject to directional selection so that 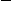 alleles are deleterious mutations having a selection differential 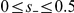 in both sexes. Mutation occurs by probability 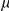 per locus per generation. Recombination occurs between 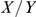 and either of the other two loci by probability 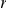 per meiosis. (Here it is assumed that 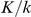 and 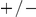 segregate independently of each other on condition of 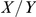, but see material S1.)

I assume that gene action is additive within loci, but the fitness consequences are multiplicative, so that the relative fitness of individual 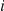 is
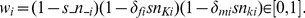
(1)


In the individual-based simulations, this is the expected relative number of offspring. Above, 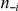, 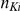 and 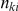 are the numbers of the respective alleles in the sex chromosomes of 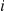, whereas 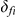 and 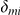 are the indicators of the phenotypic sexes (denoted by 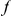 and 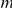).

Biologically, the proportions of phenotypic females in different genetic sexes (denoted by 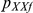, 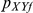 and 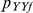) depend on each other [31]. Unless otherwise noted, it is assumed in the rest of this study that 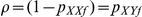, i.e. sex reversal is equally likely for both genotypic sexes. According to analysis of Grossen et al. [31], it is likely that 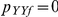 in this case, which is assumed. This makes it possible to specify the amount of sex reversal as a scalar rate 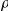. (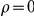 produces a pure genetic sex-determination system, and 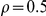 produces a pure environmental sex-determination system. In many species, values of 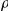 differing significantly from zero are observed [20], [23], [32], [33], [34].)

Initially, all 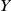 chromosomes are 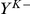 haplotypes, and all 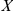 chromosomes are 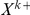 haplotypes, and the genetic sex ratio is even (i.e. 25% of all sex chromosomes are 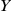 chromosomes). Locus 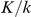 has been subject to sexual-antagonistic differentiation, whereas the fixation of the deleterious mutation represents the degeneration of the 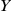 chromosomes. In this study, I investigate how undergoing sex reversal helps a population to rejuvenate its 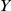 chromosomes, i.e. to purge deleterious mutations. Except for Equation 1, this framework has been directly adopted from Perrin [10].

### Dynamics of allele frequencies

I now consider how the allele frequencies evolve in time, given the framework stated above. To this end, I compose a system of difference equations. In this section, I give the equations, whereas discussion on their coefficient matrices is included in the material S1, and the matrices themselves are given in material S3. The key idea is that it is possible to model this biological system by using matrix algebra, because there are a finite number of haplotypes in the modeling framework. However, selection operates on the ‘phenotype’ of the individual which in this case comprises the phenotypic sex (two options, 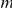 and 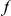), in addition to the diploid haplotype (64 options, e.g. 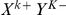). Therefore, it is necessary to consider the distribution of these phenotypes in each generation 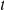, denoted by 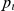. Two of the phenotypes are always biologically equivalent (e.g. 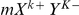 and 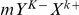), but an artificial distinction has to be made between them to model the union of gametes at a later stage (Equation 2). Out of 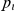, the frequencies of all alleles and the sex ratio can be calculated.

First, selection and recombination change the frequencies of the phenotypes by matrix multiplication:
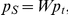


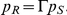



Matrix 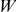 has values corresponding to selective pressure, and matrix 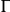 has values corresponding to probability of recombination. Then, gametes are produced. For clarity, I consider the allele frequencies of eggs and sperm separately.
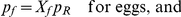


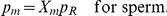



Mutation operates on 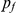 and 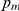. Matrix 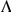 has values corresponding to probability of mutation.
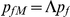


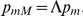



Then, union of gametes occurs. Assuming random mating, the distribution of diploid haplotypes is given by the matrix
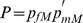
(2)


Note that Equation 2 implies non-linearity. If this was not the case, it would be a trivial task to solve the equilibrium allele frequencies explicitly. The frequencies of the phenotypes on generation 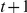 are obtained by sexing the diploid haplotypes and renormalizing the system:
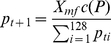
(3)


As a technical convenience, I use a mapping 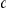 that transforms 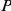 into a vector, column by column. In the material S2, there is an R code to calculate 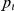 given an arbitrary initial state 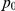.

### Individual-based simulations

The above system of difference equations is essentially a model of an infinite population. Individual-based simulations are needed for three things: to test the predictions of this theory, to investigate drift effects in the allele frequencies of small populations, and to study the population-dynamic consequences of sex reversal. To this end, I consider organisms with discrete, non-overlapping generations. The life history of these organisms is simulated by the following algorithm:

1. In each generation, the phenotypic sex of each individual is randomized. Depending on the respective genotype, an individual is a phenotypic female by probability 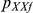, 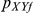 or 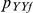, i.e. sex reversal happens at this stage.
2. The relative fitness of each individual is calculated by using Equation 1.
3. Natural selection and breeding occur. Here, I consider two options.
  - Survival selection: individual 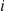 dies with probability 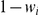 before producing offspring, but each surviving female gets Poisson(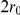) offspring. A random mate is assigned for each female from the surviving males. In this case, the expected number of offspring for individual 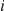 is 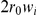.
  - Fecundity selection: the total number of offspring produced depends on all 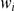 values, but no individual dies before getting chance to reproduce. Namely, each phenotypic female 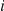 is first assigned a random mate 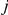 among the phenotypic males. They get Poisson(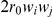) offspring, depending on their relative fitness values. In this case, the expected number of offspring for individual 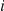 is 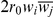 (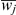 denoting the average of 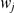 over the other sex), which is proportional to 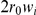.
4. Gametes are produced. In phenotypic females, recombination occurs by probability 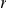 between each pair of loci. Mutations occur by probability 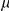 per locus per generation in both sexes. Each offspring gets one random gamete from the sire, and one random gamete from the dam. I assume that 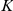 and 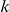 alleles may mutate into one other, but 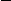 alleles cannot change into 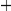 alleles.
5. The reproducing individuals are removed, and the procedure is carried out for a new generation. If population exceeds a carrying capacity 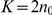 after reproduction, individuals are deleted by random. This is merely a technical convenience to avoid simulating very large populations; natural selection occurs on step 3.

The R codes used for these simulations are given in the online material S2.

### Parameter values and sensitivity analysis

Table 1 lists the relevant parameters. Their values are more or less arbitrary, as they are only meant to illustrate the range of factors that affect the dynamics of the 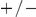 locus. A sensitivity analysis is performed by giving each parameter higher and lower values. For 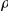, a number of values from 0 to 0.50 are tried in each scenario. Apart from changing the parameter values, a few structural assumptions are tested, namely: the effect of the carrying capacity 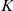, symmetric sex reversal (i.e. assumption that 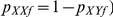, and the pattern of recombination (i.e. that segregation of 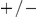 and 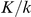 is independent on condition of 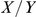). The results of sensitivity analysis are discussed in detail in material S1 and the accompanying Figures S1, S2, S3, S4, S5. Shortly, as is to be expected, selective pressures 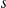 and 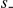 determine the advantage related to sex reversal (see below, ‘Individual-based simulations’). Secondly, the advantage related to sex reversal is lesser, if the 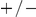 locus is in a closer linkage with the sex-determining locus, yet it is still observable.

**Table 1:** Summary of model parameters.

Parameter	Baseline value	Lower value	Upper value	Comments
Per locus rate of mutation 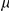	10^−4^	10^−5^	10^−3^	Applies on loci 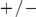 and 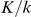
Probability of recombination 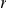	0.25	0.10	0.50	Upper value implies no molecular linkage
Rate of sexual-antagonistic selection 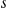	0.05	0.02	0.10	Lower value implies no fitness cost for sex reversal
Rate of directional selection 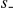	0.05	0.02	0.10	Lower value implies a neutral mutation
Initial population size 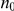	1,000	200	5,000	Applies only on finite populations
Optimal growth rate 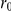	1.10	1.00	1.20	Maximal expected growth rate; used to scale the fitness in finite populations

## Results

### Dynamics of allele frequencies

The system of equations laid out above is nonlinear, because the union of gametes (Eq. 2) follows a mass-action principle. Because of this, it is possible that multiple equilibria exist. I investigated that possibility by initializing the system with random initial values (i.e. initial frequencies 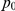), but only one equilibrium was observed for each set of parameter values, having properties discussed below.

Initially, sex reversal creates a burst of polymorphism, rapidly generating new phenotypes (such as 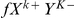) and haplotypes (such as 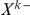). Eventually, frequencies of some phenotypes (i.e. those with the deleterious mutation) decline, as natural selection wipes them out while the system slowly approaches the equilibrium. However, all phenotypes are maintained in populations of sufficient size, because mutation and recombination always produce them.

**Figure 1:**
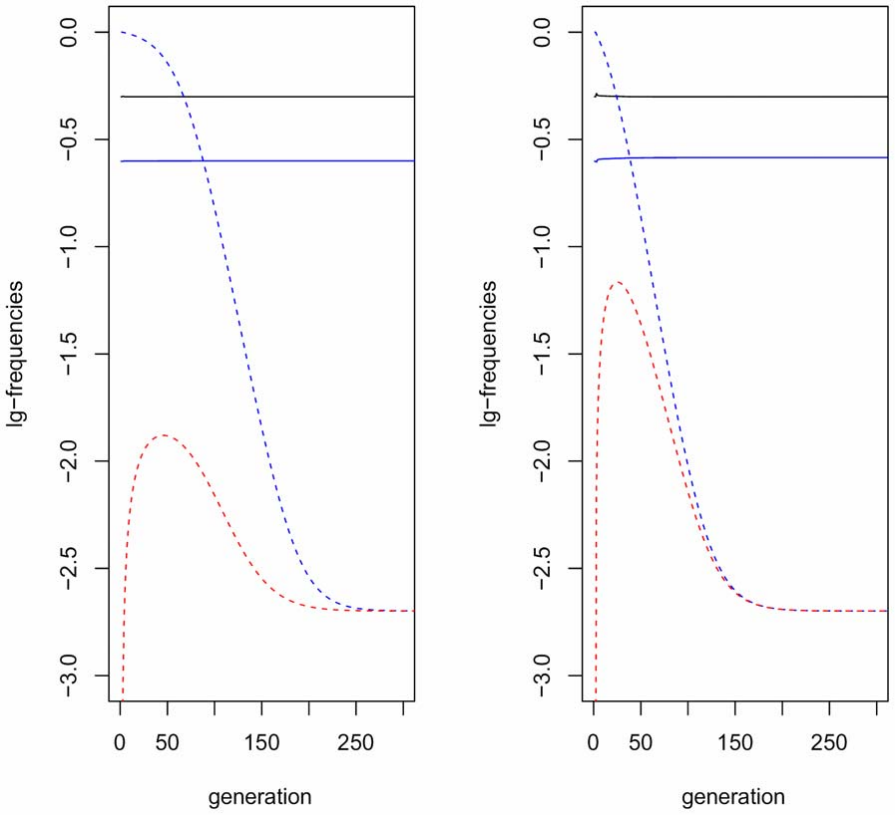
Dynamics of the deleterious mutation. In this figure, dynamics of the deleterious mutation is illustrated by two examples. In both panels, the black lines represent the proportion of phenotypic females, the blue lines the proportion of 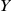 chromosomes out of all sex chromosomes, the dashed blue lines prevalence of the deleterious mutation in 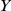 chromosomes, and the dashed red lines prevalence of the deleterious mutation in 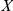 chromosomes. (Note the 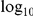 scale of the y axis.) The rates of sex reversal are 0.01 (panel A) and 0.10 (panel B). The rest of the parameters are as in ‘Baseline’, Table 1.

Figure 1 illustrates the dynamics of a few key statistics, namely the sex ratio, proportion of 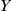 chromosomes, and prevalence of the deleterious mutation in 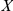 and 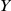 chromosomes. As can be seen from Figure 1, the deleterious mutation is purged so that the prevalence in 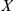 chromosomes first catches up with that of 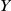 chromosomes, and then both prevalences start to approach their equilibrium value. The end result of this process is the usual mutation-selection balance, i.e. roughly 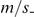, and it does not depend on 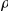. After an initial disturbance (barely visible in Fig. 1B), proportion of 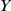 chromosomes and the sex ratio remain constant. This was to be expected on basis of earlier research: using difference equations, Grossen et al. [18] have shown that this is the equilibrium state for 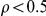, which they interpret as sex-ratio selection. However, this concerns the sex ratio after birth, given by 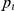, not that of adult life stage which is given by 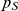; as long as there are more 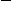 in 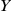 chromosomes, there are less breeding males than females in 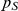.

**Figure 2:**
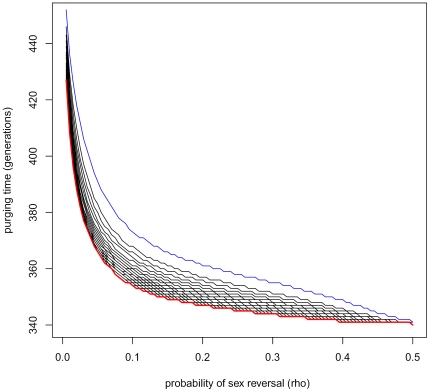
Purging time. This figure illustrates the joint effect of the probabilities of sex reversal (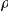) and recombination (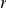). The probability of sex reversal can be read from the x axis, whereas each curve corresponds to a specific probability of recombination from 0.1 (the blue line) to 0.5 (the red line) with even spaces. Other parameter values are as in the baseline scenario of Table 1. On the y axis, the ‘purging time’ is the number of generations that is required for the allele frequencies to reach the mutation-selection balance by 10^−8^.

Higher values of 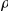 always imply that the mutation can be purged more rapidly. Using the parameter values in Table 1 and 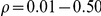, it takes 167 to 452 generations for the deleterious mutation to reach the mutation-selection balance by 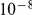 accuracy, except of the case of neutral mutation (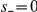) where more than 3,000 generations are required to break the linkage between 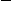 and 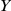. Most of the advantage is produced by relatively low values of 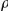 (see Fig. 2). As a rule of thumb, it could be said that 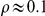 purges mutations almost as effectively as a pure environmental sex-determination system (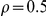).

Finally, linkage disequilibrium is a necessary condition for the fountain-of-youth hypothesis to work in this modeling framework. If the system is initialized at Hardy-Weinberg equilibrium, or by even phenotypic frequencies, sex reversal does not affect the dynamics of 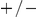 locus in any way. Because no epistasis has been assumed, recombination only helps to break the linkage disequilibrium between 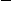 and 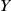. If this disequilibrium does not exist initially, recombination is not relevant, and hence sex reversal is not relevant.

### Individual-based simulations

**Figure 3:**
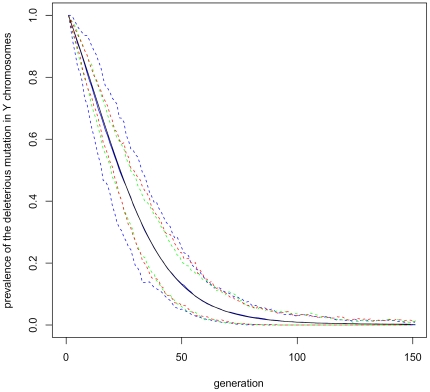
Drift effects under survival selection. Drift effects are studied in populations undergoing survival selection (Option a. in the main matter), using prevalence of the deleterious mutation in 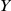 chromosomes as a summary statistic. The black line represents the theoretical value of this statistic, calculated by applying Equation 3 iteratively. The colored lines illustrate the distribution of this statistic in small populations (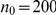 blue, 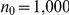 green, and 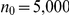 red). The dashed lines indicate the 0.05 and 0.95 quantiles of these distributions. The solid blue line indicates the sample mean for 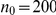. (For 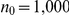 and 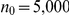, the sample mean has been omitted, because it is so near to the expectation.) For each 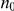, 150 Monte Carlo replicates have been generated. The rate of sex reversal is 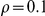, and the other parameter values are as in ‘Baseline’, Table 1.

**Figure 4:**
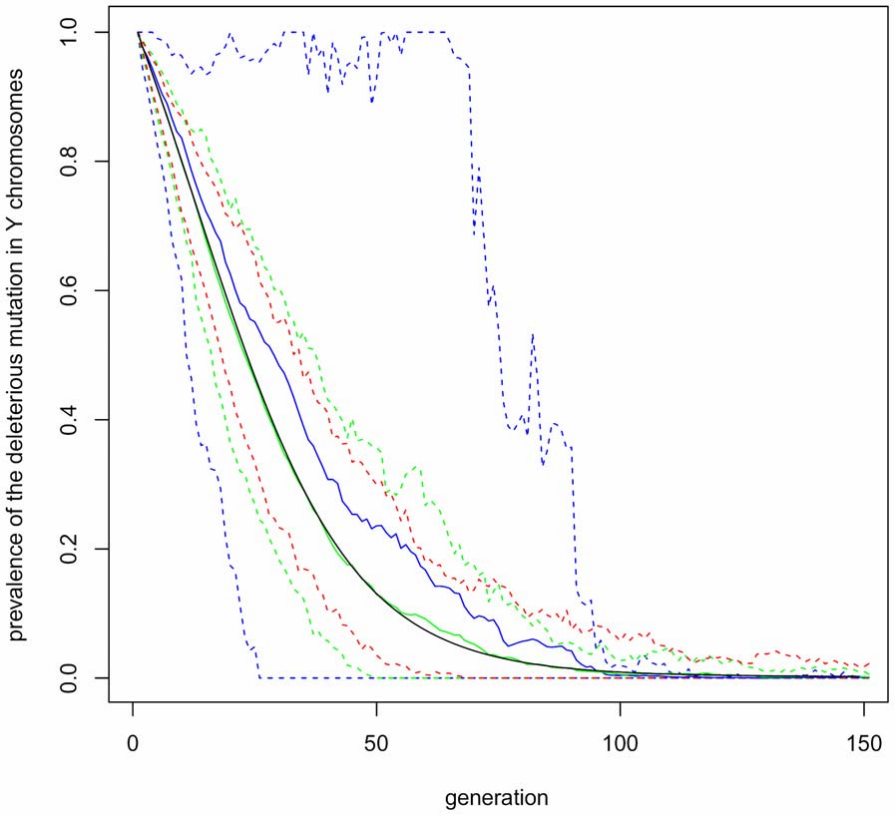
Drift effects under fecundity selection. This figure illustrates drift effects in populations undergoing fecundity selection (Option b. in the main matter), presenting the prevalence of the deleterious mutation in 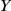 chromosomes. The black line represents the theoretical value of this prevalence, calculated by applying Equation 3 iteratively. The colored lines illustrate the distribution of this statistic in small populations (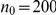 blue, 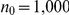 green, and 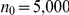 red). The dashed lines indicate the 0.05 and 0.95 quantiles of these distributions. The solid colored lines indicate the sample mean for 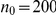 and 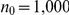. (This has been omitted for red, i.e 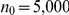, because it is so near to the expectation.) For each 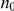, 150 Monte Carlo replicates have been generated. The rate of sex reversal is 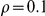, and the other parameter values are as in ‘Baseline’, Table 1.

The drift effects are illustrated by Figures 3 and 4, using the prevalence of the deleterious mutation in the 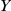 chromosomes as a summary statistic. Regardless of the drift effects, even populations of the modest initial size 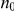 = 200 behave in great consistency with the theory developed above in ‘Dynamics of allele frequencies’. However, the drift effects are more visible for any initial population size if fecundity selection is assumed. Under survival selection, the fate of individual's genetic material depends on its own fitness, only. Under fecundity selection, the expected number of offspring depends also on the fitness of a random mate. This creates a hitch-hike type opportunity for the deleterious mutations. In some simulation runs, it was observed that the random drift drove 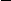 back to fixation in the 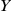 chromosomes under fecundity selection, which was never observed under survival selection.

**Figure 5:**
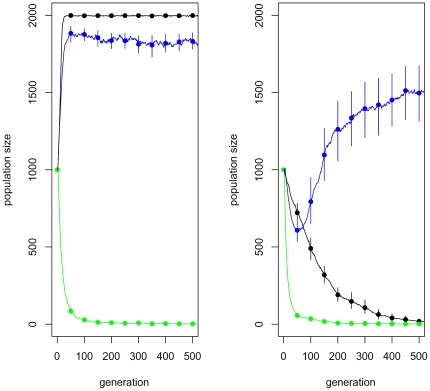
How sex reversal affects the population size. In panel A, the 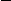 allele is neutral. In panel B, the 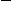 allele is mildly deleterious (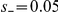), with other parameters as in ‘Baseline’, Table 1. Fecundity selection (Option b.) is assumed. The dots are estimates of the expected population size, whereas the error bars represent their uncertainty due to sampling error (95% confidence intervals). The black dots are calculated for a population with no sex reversal (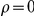), the blue dots for a moderate rate of sex reversal (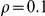), and the green dots for a pure environmental sex-determination system (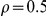). A sample of 150 populations has been used in each case.

Under fecundity selection, sex reversal has a non-uniform effect on the expected population size. This is illustrated by Figure 5. If the 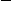 allele is neutral (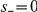), sex reversal always decreases the expected population size, because it only breaks down the co-adaptation of the 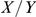 and 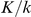 loci. For 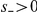, there is a trade-off between this effect and the need to purify deleterious mutations. This is shown in Figure 4B by using ‘Baseline’ parameter values. In this case, an intermediate value of 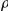 is optimal, whereas pure genetic (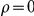) and pure environmental (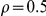) sex-determination systems decrease the expected population size. However, it should be added that due to demographic stochasticity, even populations with a nearly optimal 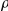 will sometimes drift into extinction.

It should be stressed that the advantage related to sex reversal concerns only fecundity selection (Option b.) and a suitable range of parameter values. I was unable to find parameter values for which sex reversal would have a significant positive effect on the expected population size under survival selection (Option a.). This is understandable by recalling the mechanism of survival selection: in Option a., the number of offspring depends only on the number of surviving females, as long as there is at least one male to breed with. In this case, it is presumably optimal to keep the 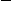 and 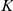 alleles out of the 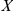 chromosomes, even if it means that the deleterious mutation cannot be purged.

## Discussion

I have used Perrin's framework [10] to study his fountain-of-youth hypothesis. According to theoretical considerations, environmental sex reversal helps to purge deleterious mutations out of the 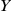 chromosomes. This was also observed in individual-based simulations for a wide range of parameters. The fitness consequences of this effect depend on the type of natural selection used in the simulation model: For populations undergoing pure survival selection, sex reversal seems to be always harmful, but for populations undergoing fecundity selection, intermediate rates of sex reversal could be optimal. Doubtless, other selection mechanisms than these two could be implemented, but the key seems to be that in survival selection, the number of males is not important, whereas in fecundity selection, male and female fitness are equally important. In short, it is only desirable to rejuvenate the 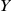 chromosomes, if the males matter.

The concept of sex reversal deserves to be discussed in some length. As far as this study is concerned, it comprises both environmental sex determination and sex changes before reproductive age. Bull [23] distinguishes between these two, so that in environmental sex determination, sex is permanently fixed as a result of environmental conditions, e.g. temperature, during ontogeny. Ah-King and Nylin [21] have proposed that phenotypic sex could be seen as a reaction norm. In that context, sex reversal can occur in all species, but some species are extremely insensitive to environmental conditions (genetic sex-determination system), whereas some other species are infinitely sensitive to them (environmental sex-determination system). Pure environmental and pure genetic sex-determination systems can be seen as opposite ends of a spectrum, with many species falling somewhere in between [21], [31], [35]. The intermediate species could still be said to have sex chromosomes, whereas pure environmental sex determination implies that no such chromosomes exist. Such is the case of many crocodilian species [22].

From the biological side, viability of the population does not imply that a trait such as sex reversal is an evolutionary advantage for the individual. This relates to the discussion about group selection [36]. In our context, turning sex reversal on in an individual-based simulation does not imply that it would evolve in nature out of the need to rejuvenate 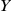 chromosomes. The next step would be to endogenize the evolution of the sex-determination system. This could be done by including a fourth locus in the present framework, one that determines liability for sex reversal, and studying its allele frequencies. Perrin also hints at this [10].

Finally, this study is insufficient to judge the truth of Perrin's hypothesis. It is almost self-evident that sex reversal helps to purify deleterious mutations out of 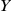 chromosomes, if recombination of sex chromosomes occurs only in phenotypic females. I have only verified the quantitative consequences of this notion. The evolutionary consequences are more complicated, as they depend on the type of selection that the species undergoes. Judging the importance of fountain-of-youth hypothesis (or more appropriately, ‘fountain-of-youth effects’), calls for studying sex reversals and recombination rates, but also the selective pressures acting in the wild.

## Supporting Information

Material S1 **Further details of the model.** In this section, further details of the model are given. Firstly, the coefficient matrices of ‘Dynamics of allele frequencies’ are discussed. Secondly, results of the sensitivity analysis are discussed.
(DOC)

Material S2 **The R programs.** In this .zip file, the R programs used for this study are given. Futher information is available in a readme file within the .zip file.
(ZIP)

Material S3 **The coefficient matrices.** In this Excel file, the coefficient matrices from ‘Dynamics of allele frequencies’ are given in their full extent, in a symbolic form.
(XLS)

Figure S1
**The effect of uneven sex reversal.** The black lines represent the proportion of phenotypic females, the blue lines the proportion of 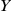 chromosomes out of all sex chromosomes, the dashed blue lines prevalence of the deleterious mutation in 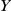 chromosomes, and the dashed red lines prevalence of the deleterious mutation in 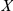 chromosomes. (Note the 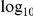 scale of the y axis.) In panel A, it is assumed that 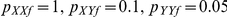, i.e. the female phenotype is strongly favored so that even some of the 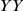 individuals are female. In panel B, the male phenotype is favored so that 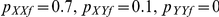.(TIF)

Figure S2
**Less favorable sex reversal under fecundity selection.** This figure illustrates the sensitivity of the results towards the rates of directional selection (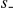) and recombination (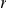). In panel A, 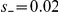. In panel B, 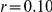. The other parameters are as in ‘Baseline’, Table 1. Fecundity selection has been assumed. The black data are for population with no sex reversal, the green data for populations with environmental sex determination, and the blue data for 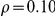. The dots and the continuous lines represent the sample mean, and the error bars represent 95% confidence intervals. Both of these figures should look like Figure 5B, if the results were not sensitive to 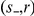.(TIF)

Figure S3
**The effect of recombination pattern.** In Figure 1, it is implicitly assumed that the chromosomal organization is 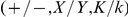, i.e. that loci 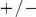 and 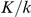 are conditionally independent. In this figure, that assumption is relaxed by modifying the recombination matrix 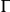. In panel A, the chromosomal organization is 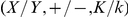, and in panel B, it is 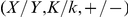. In both panels, the black lines represent the proportion of phenotypic females, the blue lines the proportion of 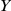 chromosomes out of all sex chromosomes, the dashed blue lines prevalence of the deleterious mutation in 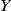 chromosomes, and the dashed red lines prevalence of the deleterious mutation in 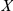 chromosomes.(TIF)

Figure S4
**The recombination pattern matters for phenotypes.** In this figure, the effect of chromosomal organization is illustrated by investigating the dynamics of phenotypic frequencies. In panel A, the chromosomal organization is 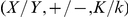, and in panel B, it is 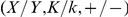. The blue lines are the frequencies of the 64 male phenotypes, and the red lines are the frequencies of the 64 female phenotypes. The parameter values are as in the ‘Baseline’ scenario, Table 1.(TIF)

Figure S5
**The recombination pattern and population dynamics.** This figure illustrates the sensitivity of the population-dynamic consequences of sex reversal towards the chromosomal organization. In panel A, the chromosomal organization is 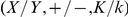, and in panel B, it is 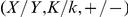. Fecundity selection has been assumed, and the parameter values are as in ‘Baseline’, Table 1. If the chromosomal organization did not matter, both of these figures should look like Figure 5B.(TIF)
