# Supplementary material for: Dynamics of a Sex-Linked Deleterious Mutation in Populations Subject to Sex Reversal
Source: PLoS One. 2011 Oct 10;6(10):e25362. doi: 10.1371/journal.pone.0025362 (PMC3189978; doi:10.1371/journal.pone.0025362)
Supplement: Material S1 — Further details of the model. In this section, further details of the model are given. Firstly, the coefficient matrices of ‘Dynamics of allele frequencies’ are discussed. Secondly, results of the sensitivity analysis are discussed. (DOC) [file pone.0025362.s001.doc]

# Supplementary material S1

### Coefficient matrices

Below, I discuss the coefficient matrices of the equations from ‘Dynamics of allele frequencies’. I acknowledge that this discussion is not rigorous. In other words, I cannot prove that my set of equations is the best one. On the other hand, it gives results that are highly consistent with individual-based simulations (see Figures 3 and 4), and some of the logic is very simple. In principle, the same set of equations can be used to model any set of genes in sexually reproducing organisms, as long as a finite number of alleles is assumed, whereas the values and dimensions of matrices $W,\Gamma,X_{f},X_{m},\Lambda$and $X_{mf}$ are specific to each problem. Most importantly, simulating the dynamics of allele frequencies can be done just by applying Equation A1 repeatedly, which is computationally efficient.

All entries of matrices $W,\Gamma,X_{f},X_{m},\Lambda$and $X_{mf}$are given in the supplementary material S3. Here, I discuss in some length the logic behind them. Firstly, selection matrix $W\in M(128\times128)$ is a diagonal matrix such that

$$w_{ii}=(1-s\_n\__{i})(1-\delta_{fi}sn_{Ki})(1-\delta_{mi}sn_{ki})\in\left[0,1\right].$$

In other words, $w_{ii}$ is calculated in direct analogy to Equation 1 in the main matter. In the individual-based simulations, the expected number of offspring for each individual is proportional to $w_{i}$, whereas in this case, the relative number of gametes that individuals of each phenotype produce is proportional to $w_{ii}$.

Entries of recombination matrix $\Gamma\in M(128\times128)$ are determined by the principle that $\Gamma_{ij}=\delta_{f}\pi_{ij}$ where $\delta_{f}$ is the indicator of female phenotype, whereas $\pi_{ij}$ is defined as the probability that the diploid haplotype of $j$ changes into that of $i$ in course of a female meiosis, and the diagonal entry $\pi_{ii}=1-\sum_{j\neq i} \pi_{ij}$ is the probability of not changing. The probabilities $\pi_{ij}$ and $\pi_{ii}$ are determined by the basic recombination rate $r$. For example, change from $fX^{k+}Y^{K+}$ to $fX^{K+}Y^{k+}$ occurs by rate $r$.

Matrices $X_{f},X_{m}\in M(8\times128)$ are merely design matrices that ‘check’ how many gametes of each type each phenotype can produce in one meiosis, formally $(X_{f})_{ij}=\delta_{fj}\Phi_{ij}$, $(X_{m})_{ij}=\delta_{mj}\Phi_{ij}$ where $\Phi_{ij}$ denotes the number of haploid haplotypes of type $i$ in the genome of phenotype $j$, and $\delta_{fj},\delta_{mj}$ are the indicators of phenotypic sexes for phenotype $j$.

In analogy to recombination, mutation can be modeled as a linear transformation, because there is a finite number of haplotypes. In mutation matrix $\Lambda\in M(8\times8)$, $\Lambda_{ij}$is defined as the probability that haplotype $j$ changes into haplotype $i$ during one generation, and the diagonal entry is the probability of not changing, i.e. $\Lambda_{ii}=1-\sum_{j\neq i} \Lambda_{ij}$.

Sexing matrix $X_{mf}\in M(128\times64)$ is in some sense analogous to matrices $X_{f}$ and $X_{m}$. It maps the frequencies of the diploid haplotypes to the frequencies of phenotypes that comprise also the phenotypic sex. Entries of $X_{mf}$ are given by the proportions of phenotypic females ($p_{..f},p_{..m}$) and the indicators of $Y$ chromosome number ($\delta_{2j}$, $\delta_{1j}$, $\delta_{0j}$) in haplotype $j$, i.e. sex reversal is modeled through matrix $X_{mf}$:

$$(X_{mf})_{ij}=\delta_{fi}(p_{XXf}\delta_{0j}+p_{XYf}\delta_{1j}+p_{YYf}\delta_{2j})+\delta_{mi}(p_{XXm}\delta_{0j}+p_{XYm}\delta_{1j}+p_{YYm}\delta_{2j}).$$

Mapping $c$ is defined as

$c(P):M(8\times8)\to\left[0,1\right]^{64}$ such that

$$c^{-1}(p_{i+8(j-1)})=p_{ij}.$$

It is merely a technical convenience that helps to avoid writing tensor equations. In summary, it holds that

$$p_{t+1}=\frac{X_{mf}c(\Lambda X_{f}\Gamma Wp_{t}p_{t}^{'}W^{'}\Gamma^{'}X_{m}^{'}\Lambda^{'})}{\sum_{i=1}^{128} p_{ti}}, (Eq.A1)$$

the apostrophe denoting transpose of the respective matrix. This is the formula used by the R code in supplementary material S2.

### Results of sensitivity analysis

In this section, I discuss results of the sensitivity analysis in more detail than it is feasible in the main matter. In general, it can be concluded that the selection differentials $s$ and $s\_$ are key to the interplay of sex reversal and the population dynamics of fecundity selection. Otherwise, the results are quite insensitive towards the assumptions made in the main matter.

Firstly, it was assumed in the main text that sex reversal is even between sexes so that $p_{XXm}=p_{XYf}=\rho$, which would be a fortunate special case in the wild. However, the results are not very sensitive towards this assumption, as illustrated by Figure S1 where the dynamics of deleterious mutation is studied under two extreme scenarios.

Concerning the parameters in Table 1, the last two ones, $n_{0}$ and $r_{0}$ do not affect the qualitative results in any way. However, a low value of $n_{0}$ implies more demographic noise and a higher extinction risk, both of which hinder observing the trends reported in the main matter. Likewise, too high (low) values of $r_{0}$ hinder observing the effect of sex reversal, because they imply that the population is always at the carrying capacity (or extinct), regardless the value of $\rho$.

The rate of mutation $\mu$ affects the results only on a very long time scale, i.e. near mutation-selection balance. The effects of $s\_$ and $r$ are illustrated in Figure S2. Intuitively thinking, a low value of $s\_$ (i.e., a low amount of $Y$ chromosomal degeneration), and a low recombination rate $r$ should make sex reversal less favourable. This is indeed observed for $s\_$ in Figure S2A. It is presumable that also in this case a low positive value of $\rho$ would be optimal, but that would be difficult to observe, because the population is near the carrying capacity even without sex reversal ($\rho=0$). This is understandable bearing in mind that $r_{0}$ is now higher in relation to $s\_$, than in the baseline results. The rate of sexual-antagonistic selection ($s$) operates analogously, but in the opposite direction than $s\_$. Concerning $r$, however, the results are unintuitive. In Figure S2B, $\rho=0.1$ is even more favourable than in the baseline scenario. This could stem from the fact that more sex reversal is needed to tap the advantage of purging $Y$ chromosomes, if the rate of recombination is low.

Concerning the structural assumptions, the deletion of individuals above the carrying capacity $K=2n_{0}$ had no observable effect on the results. In the baseline results, individuals are deleted by random until $K$ is not exceeded. The effect of this mechanism was tested by deleting individuals by probability proportional to their selection differential, i.e., by probability $\pi_{i}\propto1-\theta_{i}$.

Perhaps the most profound of the assumptions made in the main matter concerns the recombination matrix $\Gamma$, and implicitly, the chromosomal organization. In the main matter, it is assumed that $K/k$ and $+/-$ are conditionally independent. Biologically, this implies that the physical organization of the sex chromosome is $(+/-,X/Y,K/k)$, or its mirror image. Two other combinations are possible, namely $(X/Y,+/-,K/k)$ and $(X/Y,K/k,+/-)$. Results obtained under the other two combinations are illustrated by Figures S3-S5. Concerning summary statistics, dynamics resulting from the alternative combinations is surprisingly similar to the baseline case. (Compare Figures 1 and S3.) However, different chromosomal organizations induce different $\Gamma$ matrices which should show in the dynamics of the system in some way. According to Figure S4, the difference is mainly observed in the dynamics of a few male phenotypes.

Concerning coupling of the chromosomal organization and the population dynamics, the results are intuitively sound. In Figure S5, the effect of chromosomal organization is investigated by using the baseline parameter values and individual-based simulations with fecundity selection. It turns out that for $(X/Y,+/-,K/k)$, the advantage related to sex reversal shows slower than in the baseline case, but it is still observable. This was to be expected, because in this case, the deleterious mutation is in a closer linkage with the sex-determining locus than in the baseline case. Results for the chromosomal organization $(X/Y,K/k,+/-)$, however, resemble closely the baseline results presented in Figure 5.
