# Supplementary figures and images for: Dynamics of a Sex-Linked Deleterious Mutation in Populations Subject to Sex Reversal
Source: PLoS One. 2011 Oct 10;6(10):e25362. doi: 10.1371/journal.pone.0025362 (PMC3189978; doi:10.1371/journal.pone.0025362)

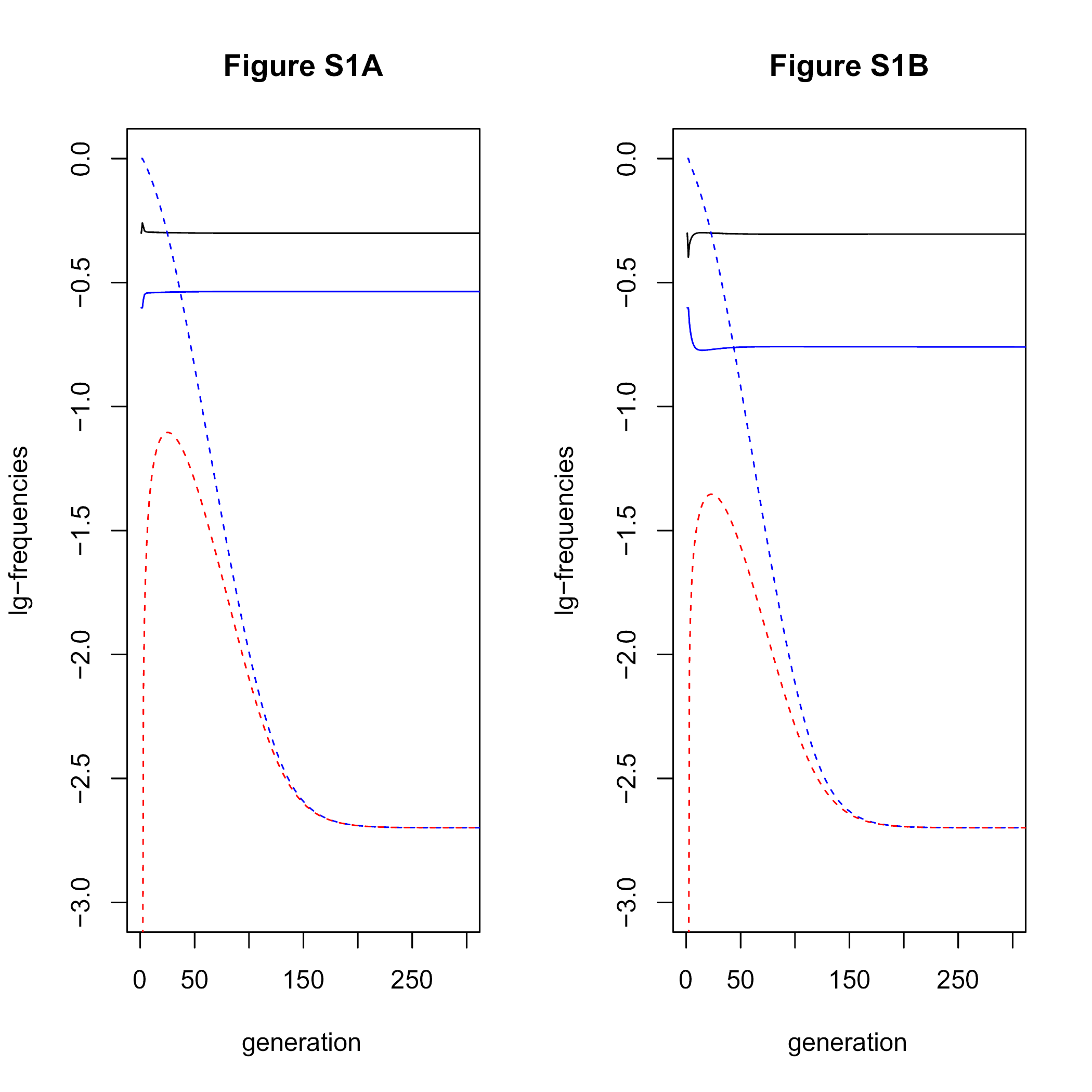

Supplement: Figure S1 — The effect of uneven sex reversal. The black lines represent the proportion of phenotypic females, the blue lines the proportion of chromosomes out of all sex chromosomes, the dashed blue lines prevalence of the deleterious mutation in chromosomes, and the dashed red lines prevalence of the deleterious mutation in chromosomes. (Note the scale of the y axis.) In panel A, it is assumed that , i.e. the female phenotype is strongly favored so that even some of the individuals are female. In panel B, the male phenotype is favored so that . (TIF) [file pone.0025362.s004.tif]

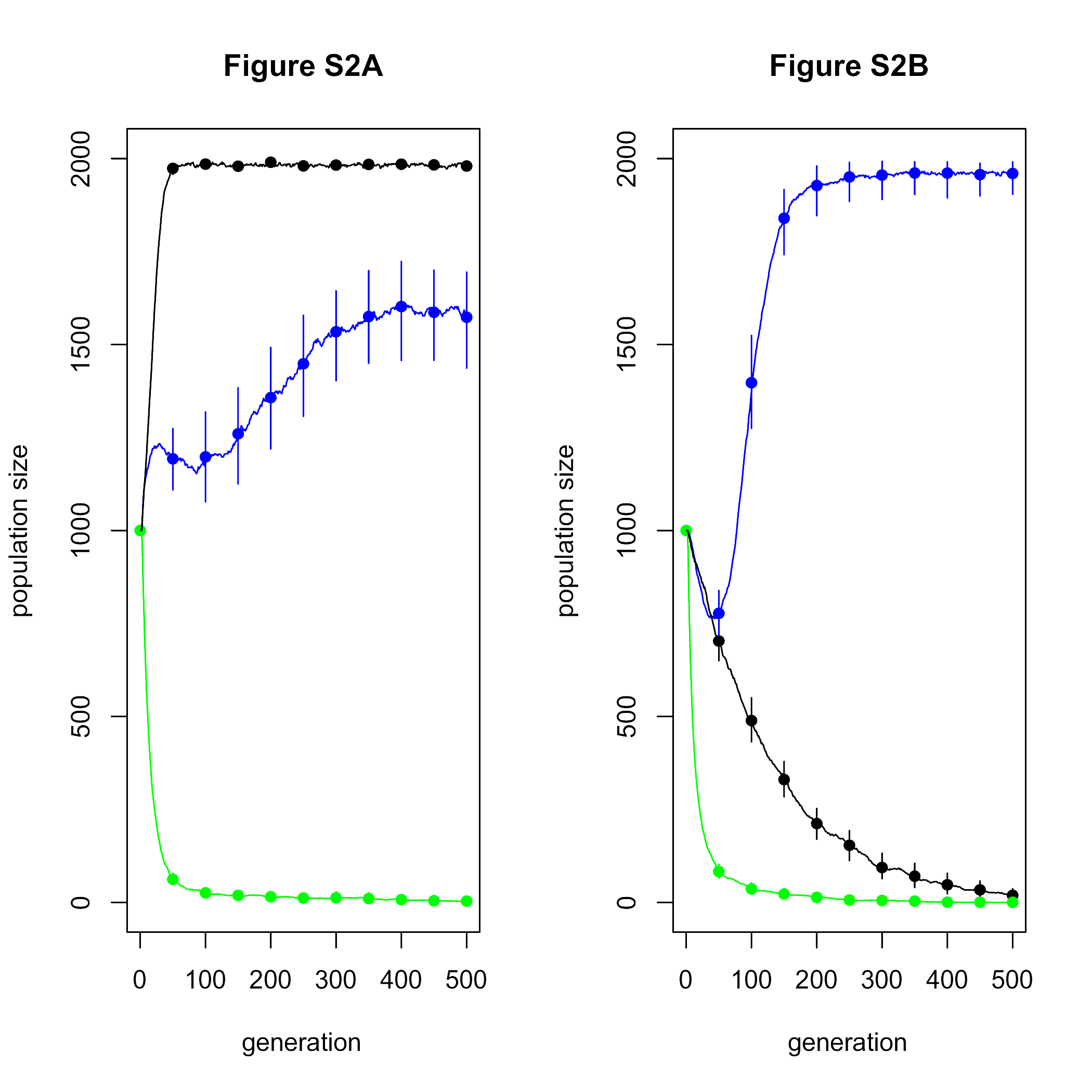

Supplement: Figure S2 — Less favorable sex reversal under fecundity selection. This figure illustrates the sensitivity of the results towards the rates of directional selection () and recombination (). In panel A, . In panel B, . The other parameters are as in ‘Baseline’, Table 1. Fecundity selection has been assumed. The black data are for population with no sex reversal, the green data for populations with environmental sex determination, and the blue data for . The dots and the continuous lines represent the sample mean, and the error bars represent 95% confidence intervals. Both of these figures should look like Figure 5B, if the results were not sensitive to . (TIF) [file pone.0025362.s005.tif]

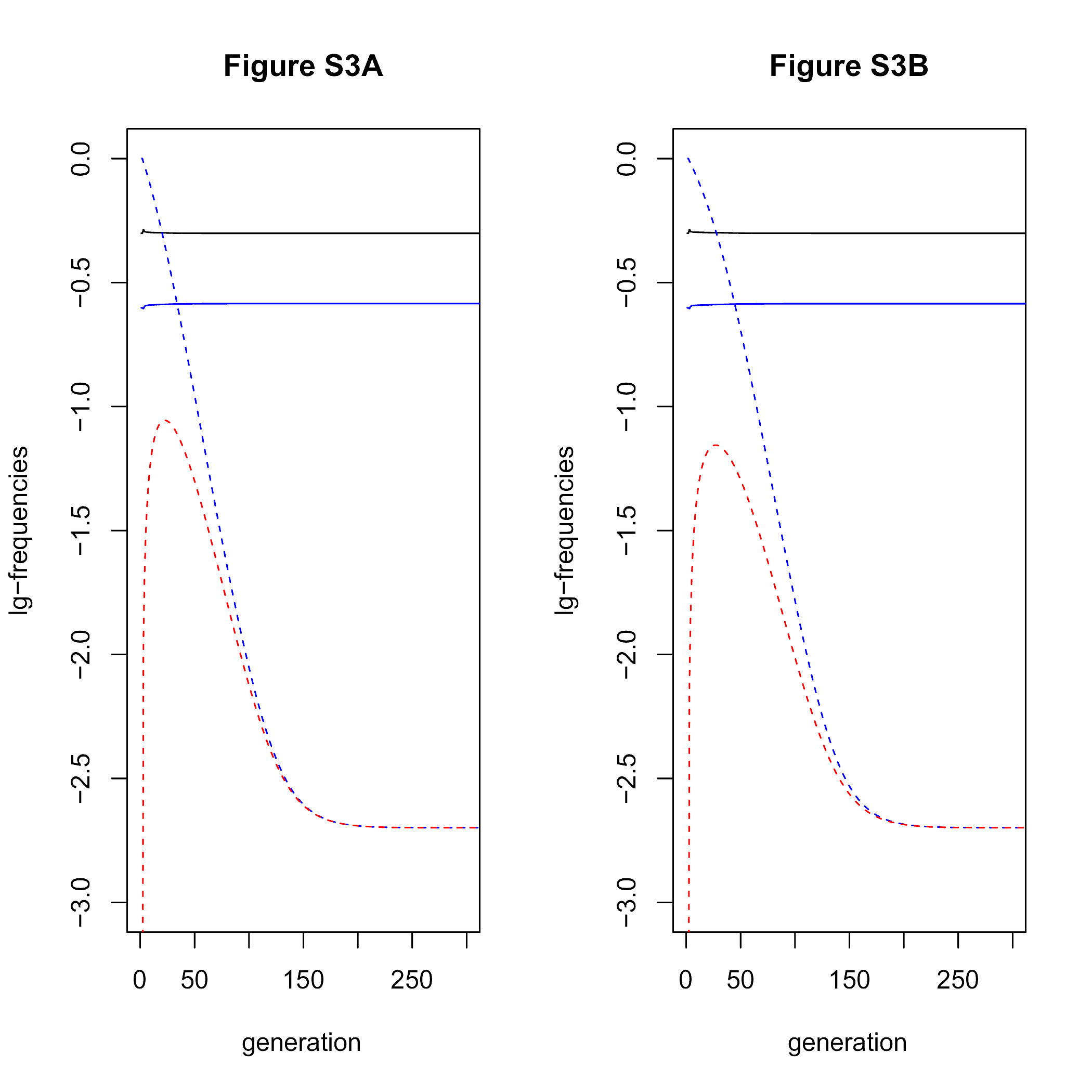

Supplement: Figure S3 — The effect of recombination pattern. In Figure 1, it is implicitly assumed that the chromosomal organization is , i.e. that loci and are conditionally independent. In this figure, that assumption is relaxed by modifying the recombination matrix . In panel A, the chromosomal organization is , and in panel B, it is . In both panels, the black lines represent the proportion of phenotypic females, the blue lines the proportion of chromosomes out of all sex chromosomes, the dashed blue lines prevalence of the deleterious mutation in chromosomes, and the dashed red lines prevalence of the deleterious mutation in chromosomes. (TIF) [file pone.0025362.s006.tif]

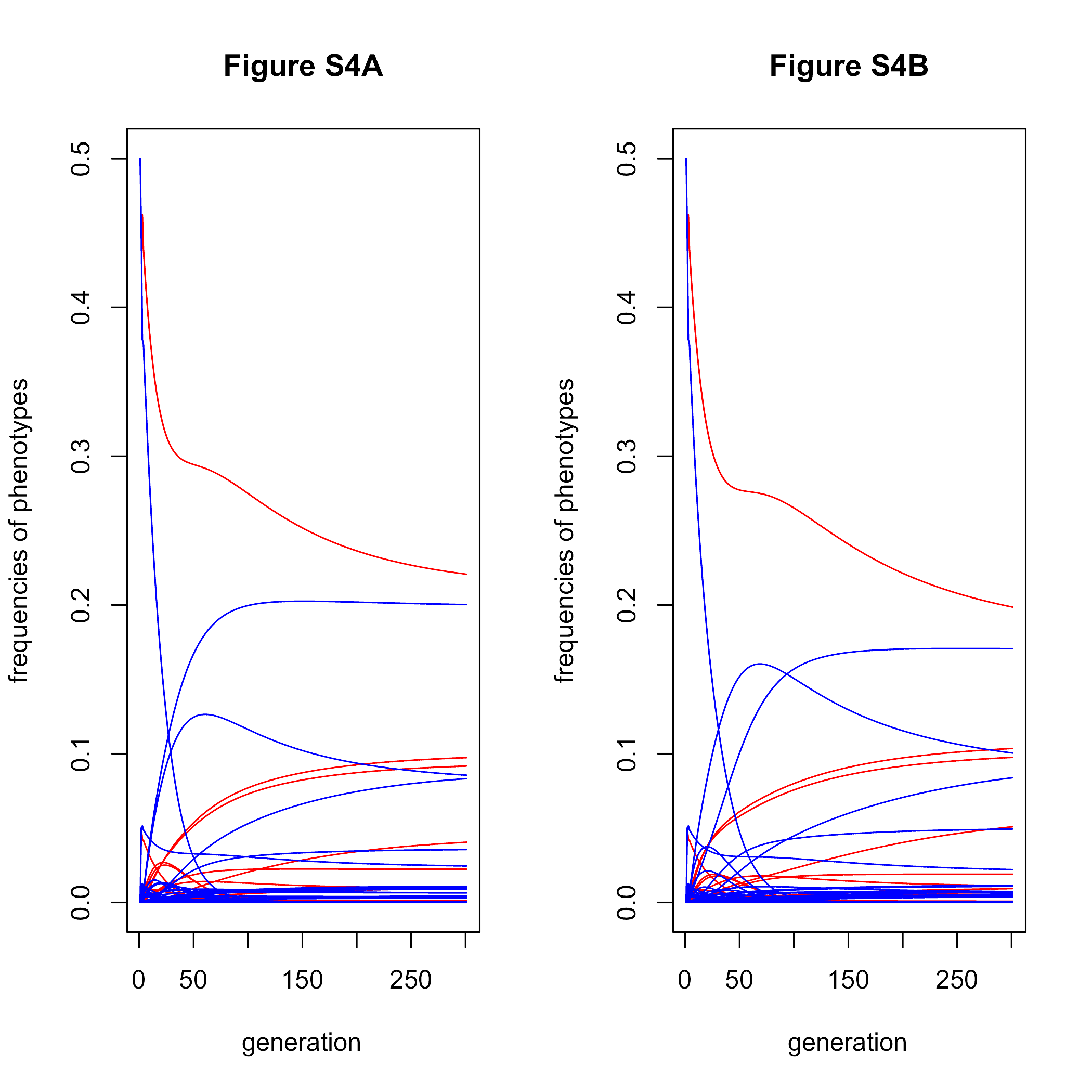

Supplement: Figure S4 — The recombination pattern matters for phenotypes. In this figure, the effect of chromosomal organization is illustrated by investigating the dynamics of phenotypic frequencies. In panel A, the chromosomal organization is , and in panel B, it is . The blue lines are the frequencies of the 64 male phenotypes, and the red lines are the frequencies of the 64 female phenotypes. The parameter values are as in the ‘Baseline’ scenario, Table 1. (TIF) [file pone.0025362.s007.tif]

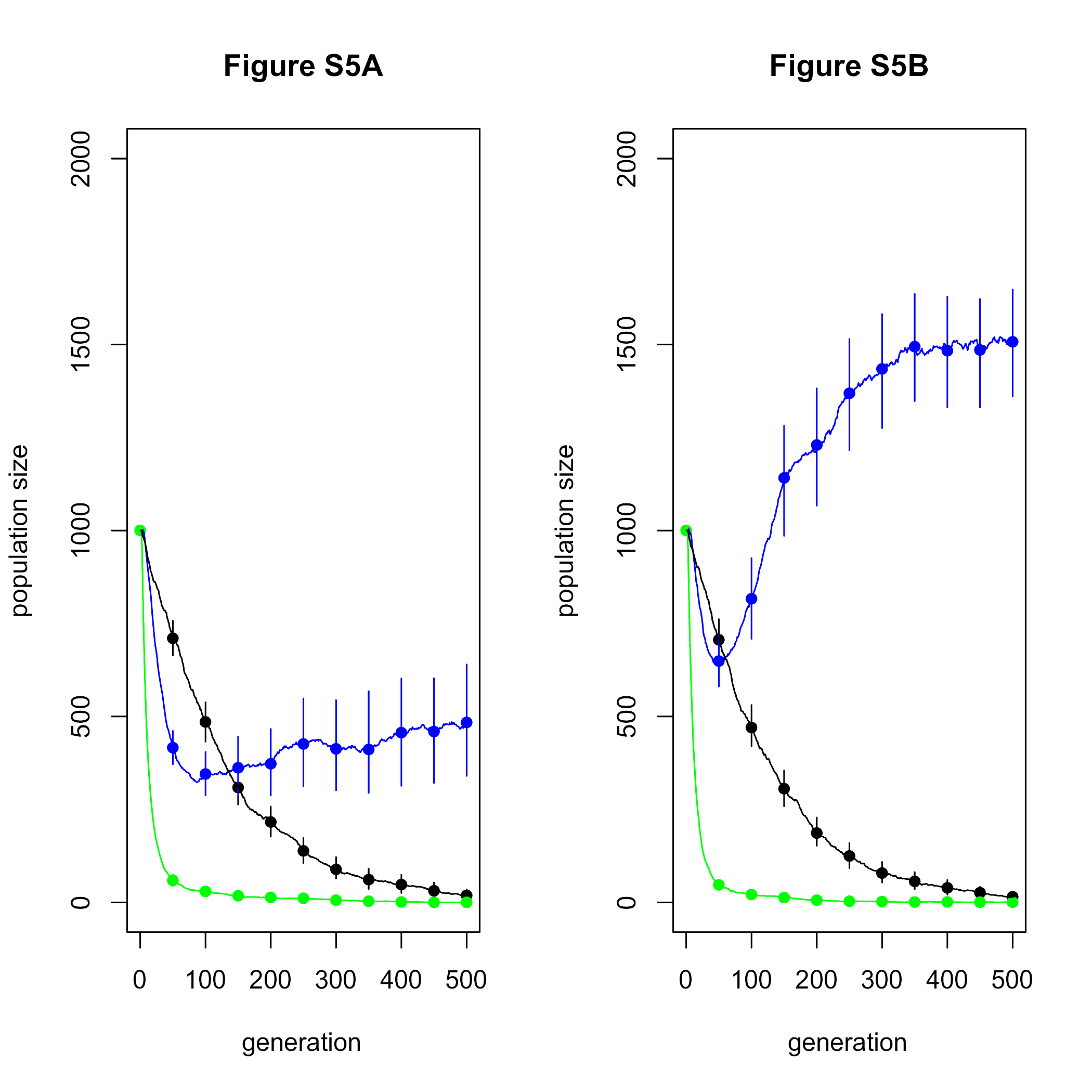

Supplement: Figure S5 — The recombination pattern and population dynamics. This figure illustrates the sensitivity of the population-dynamic consequences of sex reversal towards the chromosomal organization. In panel A, the chromosomal organization is , and in panel B, it is . Fecundity selection has been assumed, and the parameter values are as in ‘Baseline’, Table 1. If the chromosomal organization did not matter, both of these figures should look like Figure 5B. (TIF) [file pone.0025362.s008.tif]
